# Effects of Inflorescence Stem Structure and Cell Wall Components on the Mechanical Strength of Inflorescence Stem in Herbaceous Peony

**DOI:** 10.3390/ijms13044993

**Published:** 2012-04-19

**Authors:** Daqiu Zhao, Chenxia Han, Jun Tao, Jing Wang, Zhaojun Hao, Qingping Geng, Bei Du

**Affiliations:** 1 College of Horticulture and Plant Protection, Yangzhou University, Yangzhou 225009, China; E-Mails: daqiuzhao@126.com (D.Z.); hanchenxia@163.com (C.H.); wangjing3586@126.com (J.W.); haozhaojunko@126.com (Z.H.); qpgeng08@163.com (Q.G.); beiduyz@163.com (B.D.); 2 Jiangsu Key Laboratory of Crop Genetics and Physiology, Yangzhou University, Yangzhou 225009, China

**Keywords:** *Paeonia lactiflora*, mechanical strength, cloning, gene expression

## Abstract

Herbaceous peony (*Paeonia lactiflora* Pall.) is a traditional famous flower, but its poor inflorescence stem quality seriously constrains the development of the cut flower. Mechanical strength is an important characteristic of stems, which not only affects plant lodging, but also plays an important role in stem bend or break. In this paper, the mechanical strength, morphological indices and microstructure of *P. lactiflora* development inflorescence stems were measured and observed. The results showed that the mechanical strength of inflorescence stems gradually increased, and that the diameter of inflorescence stem was a direct indicator in estimating mechanical strength. Simultaneously, with the development of inflorescence stem, the number of vascular bundles increased, the vascular bundle was arranged more densely, the sclerenchyma cell wall thickened, and the proportion of vascular bundle and pith also increased. On this basis, cellulose and lignin contents were determined, *PlCesA3*, *PlCesA6* and *PlCCoAOMT* were isolated and their expression patterns were examined including *PlPAL*. The results showed that cellulose was not strictly correlated with the mechanical strength of inflorescence stem, and lignin had a significant impact on it. In addition, *PlCesA3* and *PlCesA6* were not key members in cellulose synthesis of *P. lactiflora* and their functions were also different, but *PlPAL* and *PlCCoAOMT* regulated the lignin synthesis of *P. lactiflora*. These data indicated that *PlPAL* and *PlCCoAOMT* could be applied to improve the mechanical strength of *P. lactiflora* inflorescence stem in genetic engineering.

## 1. Introduction

Herbaceous peony (*Paeonia lactiflora* Pall.) is a traditional famous flower with a cultivation history of more than 4000 years in China, which has shared the name “the king and minister of flowers” with tree peony [1]. Due to its huge flowers, bright flower colors, beautiful flower types and strong flower fragrances, it is loved by all people around the world, and expanded from the first garden cultivation to the cut flower cultivation. In Europe and the United States, herbaceous peony, known as the “wedding flower”, is a high-grade cut flower for weddings and other festive occasions, so its market prospect is very broad [2,3]. In the production practice, we find that the *P. lactiflora* inflorescence stem is easy to bend or break and can not support flower weight, which seriously affects the quality of the cut flower. Previous research reveal that mechanical strength is an important characteristic of stem, which not only affects plant lodging, but also plays an important role in the inflorescence stem bend or break [4,5]. Therefore, it is crucial to identify factors that influence mechanical strength of *P. lactiflora* inflorescence stem and study how these factors can be manipulated.

The plant cell wall, composed of cellulose, hemicellulose, lignin, polysaccharides, and proteins, is a strong fibrillar network which provides mechanical support to cells, tissues and the whole plant [6,7]. According to cell type, cellulose usually accounts for 20% to 30% of the dry weight of the primary cell wall, and constitutes 40% to 90% of the secondary wall [8]. After cellulose, lignin is the second major biopolymer component of the plant cell wall [5]. As a result, the relationship of these two components and stem mechanical strength have been popular research topics, which have been studied thoroughly in the Gramineae. For example, both culm brittleness of rice mutants (BC1, 3, 5, 7, 10) and barley mutants are caused by decreased cellulose content [6,9–14]. In addition, decreased lignin content reduced culm mechanical strength of rice flexible culm1 mutant (fc1) [5]. However, to our knowledge, there are few reports regarding ornamental plants.

Cellulose synthase (CesA) is a key enzyme of plant cellulose biosynthesis, which can catalyze the formation of β-(1,4)-linkages [12]. Even though the CesA complexes were first discovered in the plasma membrane of *Oocystis apiculata* in 1976 [15], it was not until 1996 that the first *CesA* gene was isolated in higher plant [16]. Since then, *CesA* has been identified from many plants, such as *Arabidopsis thaliana* [17], *Oryza sativa* [18], *Hordeum vulgare* [19], *Physcomitrella patens* [20] and so on. Research show that activity of *CesA* is closely related to cellulose content [13,14,21], such as in rice mutant BC7, reduced cellulose content is caused by an abnormal *CesA* gene [11]. On the other hand, a great deal of enzyme genes involved in the lignin biosynthetic pathway, among which, l-phenylalanine ammonia-lyase (PAL) and caffeoyl-CoA 3-*O*-methyltransferase (CCoAOMT) are two key enzyme genes. The former is the first gene of the phenylpropanoid pathway which catalyzes the formation of cinnamic acid from l-phenylalanine, and the latter catalyzes the formation of feruloyl-CoA and sinapoyl-CoA using caffeoyl-CoA and 5-hydroxyferuloyl-CoA as substrates, respectively. Moreover, transcript levels of these two genes directly affect lignin biosynthesis [22–24], which has become a hot topic in the field of lignin genetic engineering.

In this study, in order to identify factors that influence mechanical strength of *P. lactiflora* inflorescence stem and their regulatory mechanism, mechanical strength, morphological indices, cellulose and lignin contents, microstructure of inflorescence stem were measured and observed. In addition, related genes involved in cellulose and lignin biosynthetic pathway including *PlCesA3*, *PlCesA6* and *PlCCoAOMT* were isolated, and expression patterns of these isolated genes and *PlPAL* were investigated. These results could provide a theoretical basis for improving the quality of *P. lactiflora* cut flowers.

## 2. Results

### 2.1. Mechanical Strength and Morphological Indices

**Figure 1:**
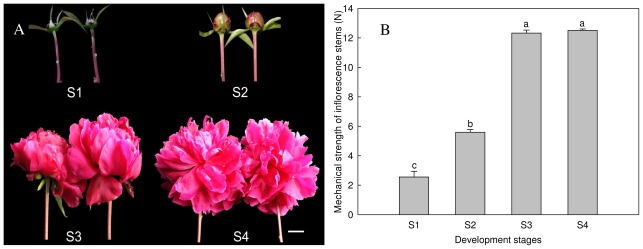
Plant phenotype and mechanical strength of inflorescence stem. (**A**) Plant phenotype of four development stages; (**B**) Mechanical strength of inflorescence stem. Different letters including a, b and c indicate significant difference at the 0.05 level. Bar = 2 cm.

Inflorescence stems of four development stages were used as materials to study impact factors of the mechanical strength of *P. lactiflora* (Figure 1A). Determination using 3-point bending-tests showed that their mechanical strength increased from S1 to S4, and S4 was 4.8 times higher than S1, but the difference between S3 and S4 was not significant (Figure 1B).

Morphological indices of plant developmental stages were shown in Table 1. All indices showed an increasing trend and reached their maximum in S4. Plant height, diameter and fresh weight of flower all reached a significant level between each stage. However, the other indices, *i.e.*, diameter and fresh weight of inflorescence stem were almost identical in S3 and S4. Correlation analysis between morphological indices and mechanical strength revealed that these five indicators showed they were positively correlated with mechanical strength, and significant level was reached by plant height, diameter of inflorescence stem, diameter of flower and fresh weight of flower. Moreover, the highest correlation was diameter of inflorescence stem (Table 2).

### 2.2. Microstructure

Microstructure of inflorescence stem was observed by optical microscope. The results showed that both the number of vascular bundles and the proportion of vascular bundle as well as pith presented an increasing trend, but a significant difference between each stage, especially S3 and S4 was not observed (Table 3).

**Figure 2:**
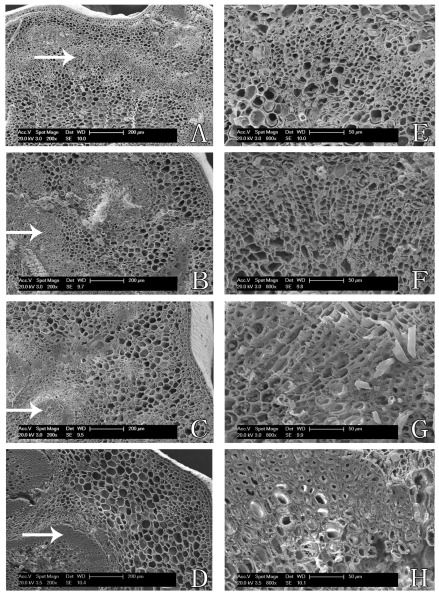
Observation of inflorescence stem microstructure with scanning electron microscope. (**A**–**D**) Photographs of development inflorescence stem with a magnification of 200 times; (**E**–**H**) Photographs of partial enlargement in A–D marked by the arrow.

In order to more intuitively observe microstructure changes of inflorescence stem, environmental scanning electron microscope was applied. Figure 2A–D showed the photographs of four development stages of inflorescence stems with a magnification of 200 times, and Figure 2E–H showed the partial enlargement of Figure 2A–D marked by an arrow. As shown in Figure 2, inflorescence stem had formed various parts in the initial stage, *i.e.*, epidermis, cortex and vascular cylinder, moreover, vascular bundles were cylindrical and close each other. With the development of the inflorescence stem, inner vascular bundles began to emerge, the cell was more closely arranged and the sclerenchyma cell wall thickened.

### 2.3. Cellulose and Lignin Contents

**Figure 3:**
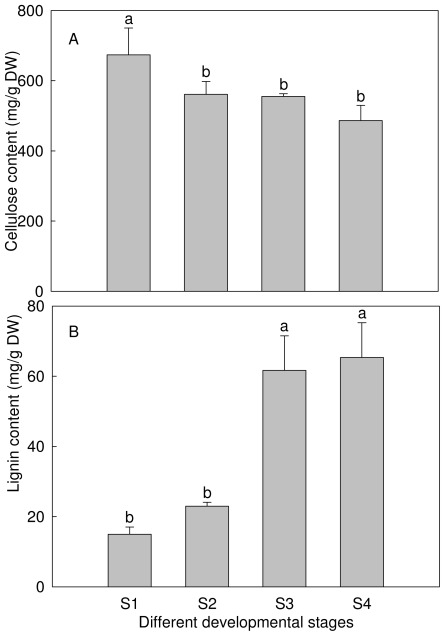
Cellulose and lignin contents of inflorescence stem. (**A**) Cellulose content of inflorescence stem; (**B**) Lignin content of inflorescence stem. ^a,b^ indicate significant difference at the 0.05 level.

As the major components of plant cell wall that play a role in mechanical support, cellulose and lignin contents were determined in these inflorescence stems. As shown in Figure 3, cellulose content had been declining from S1 to S4, but the difference between the last three stages was not significant. Its correlation coefficient with mechanical strength was −0.85. In contrast, lignin content increased gradually with the development of inflorescence stem, and S4 was 4.3 times as high as S1. This trend was the same as mechanical strength. Meanwhile, a highly significant positive correlation between them (*R* = 0.99 **) was observed.

### 2.4. Isolation and Sequence Analysis

In order to clarify the synthesis of cellulose and lignin, we aimed to isolated key biosynthetic genes. The 3′-ends of two *CesA* were obtained by 3′-RACE and were 2300 and 2015 nucleotides long. On the basis of the partial sequence, 5′-RACE was carried out to isolate the 5′-ends, which obtained a 2500 and 1800-bp fragment, respectively. Sequence splicing showed that the full-length cDNA sequence of *CesA1* was 3939 bp, and contained a whole open reading frame (ORF) of 3246 bp, an untranslated region (UTR) in 5′ end of 447 bp, a 3′-UTR of 246 bp and a complete poly A tail. *CesA2* was 3846 bp in length which contained an ORF of 3265 bp, an UTR of 410 bp in 5′ end, a 3′-UTR of 171 bp and a full poly A tail. Gene-specific primers were used for RACE of *CCoAOMT* gene, which resulted in an approximate 410 and 640-bp band of 3′ and 5′ cDNA ends, respectively. The spliced results showed that 1004 bp *CCoAOMT* cDNA contained an UTR of 57 bp in 5′ end, a 744 bp ORF, a 3′-UTR of 203 bp and a poly (A) tail.

**Figure 4:**
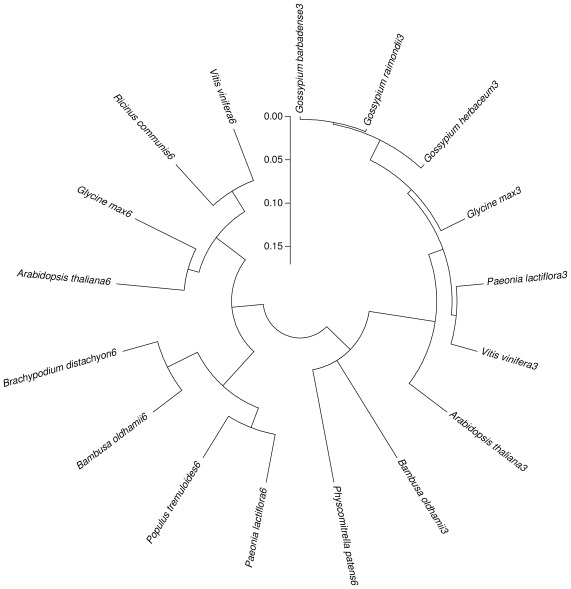
Phylogenetic tree of *PlCesA3*, *PlCesA6* and *CesA* from some other plants. The amino acid sequences were obtained from GenBank: *Arabidopsis thaliana3* (NP_196136); *Bambusa oldhamii3* (AAY43225); *Glycine max3* (XP_003533898); *Gossypium barbadense3* (ADZ16119); *Gossypium herbaceum3* (ADZ16120); *Gossypium raimondii3* (ADZ16121); *Vitis vinifera3* (XP_002278997); *Arabidopsis thaliana6* (NP_201279); *Bambusa oldhamii6* (AAY43223); *Brachypodium distachyon6* (XP_003557327); *Glycine max6* (XP_003525098); *Physcomitrella patens6* (ABI78959); *Ricinus communis6* (XP_002524299); *Vitis vinifera6* (XP_002265955).

**Figure 5:**
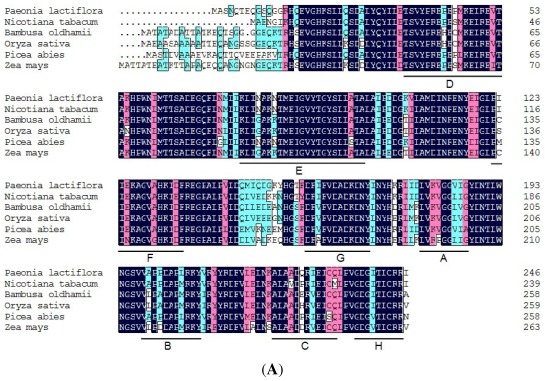

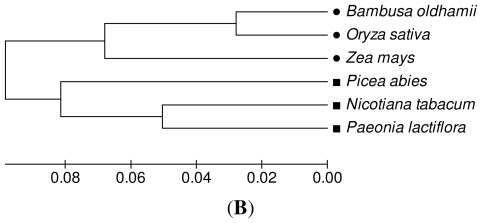
Sequence alignment and phylogenetic tree of *CCoAOMT* from *Paeonia lactiflora* other plants. (**A**) Sequence alignment; (**B**) Phylogenetic tree. The amino acid sequences were obtained from GenBank: *Bambusa oldhamii* (EF028662); *Nicotiana tabacum* (AF022775); *Oryza sativa* (AY644636); *Picea abies* (AM262870); *Zea mays* (AJ242981). Color indicates homology level, black = 100%, pink ≥75%, blue ≥50%. A, B and C are popular conserved sequence elements in plant methyltransferase; D, E, F, G and H are signature sequences of *CCoAOMT*.

*CesA1* and *CesA2* encoded 1087 and 1081 amino acids, respectively, which were 68% identical. Multiple alignment analysis indicated that *CesA1* and C*esA2* had a high homology to *CesA* from other plants, such as 88% and 67% identity with *Betula luminifera CesA* (ACJ38667), 85% and 67% identity with *Populus ussuriensis CesA* (ADV58936), 79% and 66% identity with *Hordeum vulgare CesA* (AAR29964), respectively. The phylogenetic tree that was constructed using the neighbor-joining method revealed *CesA* in these plants was divided into two categories, *CesA1* and *CesA2* in *P. lactiflora* belonged to *CesA3* and *CesA6*, respectively (Figure 4). Therefore, these two genes were designated as *PlCesA3* and *PlCesA6* with accession number JQ728998 and JQ728999, respectively. For *CCoAOMT* in *P. lactiflora*, a blast analysis showed that this protein shared 90–94% identity and 95–98% similarity with *CCoAOMT* from *Vitis vinifera* (XP_002282867), *Populus tremuloides* (AAA80651), *Broussonetia papyrifera* (AAT37172), *Populus trichocarpa* (ACC63876), *Codonopsis lanceolata* (BAE48788) and *Solanum tuberosum* (BAC23054). Amino acid sequence alignment of *CCoAOMT* in *Paeonia lactiflora* and *Bambusa oldhamii*, *Nicotiana tabacum*, *Oryza sativa*, *Picea abies* and *Zea mays* was performed, the result showed *PlCCoAOMT* had conserved sequence elements of the *CCoAOMT* gene, namely A, B, C, D, E, F, G and H (Figure 5A). Among them, A, B and C were commonly found in the plant methyltransferase gene, and D, E, F, G and H were unique in the *CCoAOMT* gene. Meanwhile, their phylogenetic tree displayed *CCoAOMT* in these plants was divided into two categories. Dicot and monocotyledons, and *Nicotiana tabacum* was the one most similar to *P. lactiflora* (Figure 5B). This coincides with the traditional plant taxonomy. Additionally, this sequence had been submitted to GenBank with the accession numbers JQ684014.

### 2.5. Expression Analysis

**Figure 6:**
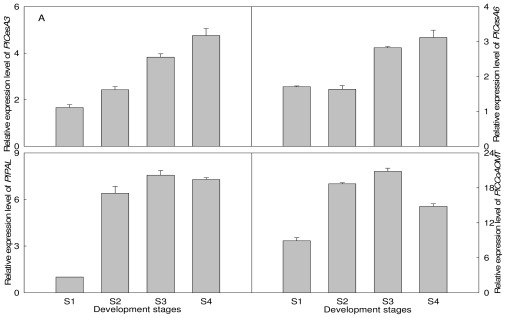

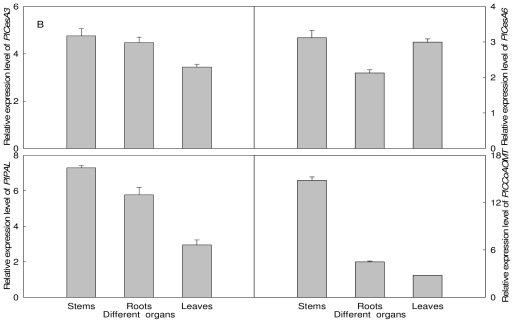
Expression levels of cellulose and lignin biosynthetic genes in tissues. (**A**) Gene expression levels in developmental inflorescence stems; (**B**) Gene expression levels in different organs.

To examine whether cellulose and lignin contents in different developmental inflorescence stems could be related to the expression of related biosynthetic genes, transcript levels of three genes isolated in this paper, *PlCesA3*, *PlCesA6* and *PlCCoAOMT*, together with *PlPAL* (JQ070801) we had isolated from *P. lactiflora,* were analyzed by real-time quantitative polymerase chain reaction (Q-PCR). This is a highly sensitive, accurate, rapid and high-throughput technique [25]. We found that the expressions of these genes could be detected in all tissues, but the expression levels were different (Figure 6). During the developmental stages of inflorescence stems, the transcript levels of *PlCesA3* and *PlCesA6* almost showed an upward trend, but the expression levels of *PlPSY* and *PlCCoAOMT* trended similarly which increased from S1 to S3, and with a little decrease in S4. In different organs, transcript levels of *PlCesA3* and *PlCesA6* were different, and their minimum consisted in leaves and roots, respectively. For *PlPSY* and *PlCCoAOMT*, the expression patterns were identical, their highest levels of transcription occurred in stems which were 156.76% and 433.25% more than those of leaves, respectively.

## 3. Discussion

Stem mechanical strength is related to the morphological indices of a plant, *i.e.*, plant height, diameter and dry weight of stem *etc.* Therefore, varieties with short plants and sturdy stems are chosen in lodging resistance breeding of crops [26,27]. In this study, we found that the correlation between mechanical strength and fresh weight of inflorescence stem did not reach a significant level, which indicated that the fresh weight of an inflorescence stem could not accurately reflect the texture density of an inflorescence stem, and could not be used as an indicator to estimate mechanical strength. In contrast, a significant level was reached by plant height, diameter of inflorescence stem, diameter of flower and fresh weight of flower. Moreover, the highest correlation was diameter of inflorescence stem. These results indicate that diameter of inflorescence stem was a direct indicator in estimating mechanical strength, and other morphological indicators also influenced it. In addition, with the development of *P. lactiflora* inflorescence stem, the number of vascular bundles increased, vascular bundles were arranged more densely, the sclerenchyma cell wall thickened, and the proportion of vascular bundle and pith also increased, which were consistent with previous studies [12,28–30].

Cellulose is the main component of the cell wall skeleton, its basic unit is microfibril, which can maintain the cell shape and enhance the mechanical strength of the plant, and therefore the cellulose content is directly related to the mechanical tissues [31]. On the other hand, lignin, which distributes in the cell wall of plant lignified mechanical and conducting tissues can increase cell wall strength, cell wall impermeability and stem mechanical strength [32]. In rice [6,9–12], wheat [4], barley [13,14], rape [33], maize [34] and other plants, their mechanical strengths are correlated with cellulose, lignin or neither of them. This indicates that the factors that influence stem mechanical strength are different for different plants, or even different mutants in the same plants. Besides cellulose and lignin, some other chemical components affect mechanical strength. For example, glucose and xylulose are positively related to the lodging resistance in rice [35,36]. In *P. lactiflora*, cellulose content decreased while the lignin content increased, but the cellulose content was much higher than the lignin. We speculated that cellulose was not strictly correlated with mechanical strength of *P. lactiflora* inflorescence stem, and lignin had a significant impact on it. Combined with microstructure of inflorescence stem, there might be other increased chemical components in the cell wall. Further study is needed.

Functional studies of *CesA* showed that it was a super gene family. The enzymes involved in cellulose synthesis were not the same in different development stages of plant cell walls [37]. In the *CesA* gene family of *Arabidopsis thaliana*, *CesA1*, *CesA3*, and *CesA6* played an irreplaceable role in the synthesis of the primary cell wall [38]. *CesA4*, *CesA7* and *CesA8* had a direct relationship with the formation of secondary cell wall [39,40]. In this study, two isolated gene members were confirmed as *CesA3* and *CesA6* in *P. lactiflora*, their expression levels were inconsistent with cellulose contents in development inflorescence stems, and their expression patterns in different organs were not the same, which indicated that these two genes were not key members in cellulose synthesis of *P. lactiflora* and their functions were also different. For *PAL* and *CCoAOMT* in *P. lactiflora*, their expression patterns were identical in all tissues. Their expression levels were greatly increased from S1 to S2 of inflorescence stem and decreased in the last stage. However, lignin content increased and a tremendous increase occurred from S2 to S3, which suggested that these two genes regulated lignin synthesis, but their transcript levels and lignin synthesis were out of sync. These results coincided with report about elephant grass [41]. These results indicated that *PlPAL* and *PlCCoAOMT* could be used to improve the mechanical strength of *P. lactiflora* inflorescence stems, which provide a basis for using genetic engineering means to improve the quality of *P. lactiflora* cut flowers.

## 4. Experimental Section

### 4.1. Plant Materials

Four development stages inflorescence stems of *P. lactiflora* cultivar “Hongyanzhenghui” were taken from the germplasm repository of Horticulture and Plant Protection College, Yangzhou University, Jiangsu Province, China (32°30′ N, 119°25′ E). After determination of mechanical strength and morphological indices in 5 cm of top inflorescence stem, one part was fixed in 3% glutaraldehyde using for microstructure observation, and the other was immediately frozen in liquid nitrogen, and then stored at −80 °C until analysis

### 4.2. Morphological Indices and Mechanical Strength Determination

Plant height was measured by meter stick (Zhejiang Yuyao Sanxin Measuring Tools Co., Ltd., Yuyao, China), fresh weight and diameter of inflorescence stem and flower were measured by balance (Gandg Testing Instrument Factory, Changshou, China) and micrometer scale (Taizhou Xinshangliang Measuring Tools Co., Ltd., Taizhou, China), respectively. In addition, mechanical strength of inflorescence stem was tested with a universal NK-2 digital force testing device (Zhejiang Hui’er Instrument & Equipment Co., Ltd., Hangzhou, China).

### 4.3. Microstructure Observation

For paraffin section, inflorescence stem fixed in 3% glutaraldehyde was washed 3 times with 0.1 mol/L phosphate buffer, and dehydrated using 30%, 50%, 70%, 85%, 95% and 100% gradient ethanol. Moreover, they passed through transparence, infiltration paraffin, embedment, section at 10 μm (Leica, Wetzlar, Germany), dewaxing and staining, and were then observed with the Zeiss the Primo Star 176045 microscope (Zeiss, Oberkochen, Germany).

For observation of scanning electron microscope, the fixed inflorescence stem was dehydrated in a gradient ethanol solution and treated with the mixtures of acetone: anhydrous alcohol (1:1, 2:1, 1:0, v/v), acetone:isoamyl acetate (1:1, 1:2, v/v), and pure isoamyl acetate. After drying and spraying gold (EIKO IB-3, Hitachi, Japan), the sample was observed by environmental scanning electron microscope (Philips XL-30 ESEM, Amsterdam, Holand).

### 4.4. Cell Wall Materials Fractionation, Cellulose and Lignin Contents Determination

The cell wall materials were fractioned according to the method of Rose *et al.* [42] with some modifications. Briefly, mature inflorescence stem of herbaceous peony was ground into fine powder in liquid nitrogen and extracted with 95% alcohol, and then washed twice with boiling alcohol and methyl alcohol:chlorination (1:1, v/v), respectively. Then, the cell wall residues were dried overnight at 30 °C. Cellulose content was measured by the anthrone [43], and lignin content was determined following the method of Müsel *et al.* [44].

### 4.5. RNA Extraction and Primers Design

Total RNA was extracted according to a modified CTAB extraction protocol used in our laboratory [45]. Prior to reverse-transcription, RNA samples were treated with DNase using DNase I kit (TaKaRa, Kyoto, Japan) according to the manufacturer’s guidelines.

3′ rapid-amplification of cDNA ends (RACE) primers were designed according to the retrieved *CesA* and *CCoAOMT* cDNA sequences of other plants from GenBank. And then on the basis of the 3′ cDNA sequences, 5′ RACE primers were designed. In gene expression analysis, the *P. lactiflora Actin* (GenBank Accession No. JN105299) was used as an internal control, and the expression analysis primers were designed according to the full-length cDNAs of isolated *PlCesA*, *PlCCoAOMT* and *PlPAL* (JQ070801). All mentioned primers were together listed in Table 4, which were all designed using DNAMAN 5.0 (Lynnon Corporation: Quebec, Canada, 1994) and Primer Premier 5.0 (Premier Biosoft International: Palo Alto, CA, USA, 2004), and synthesized by Shanghai Sangon Biological Engineering Technology & Services Co., Ltd. (Shanghai, China).

### 4.6. Isolation of the Full-length cDNA Sequence

Isolation of cDNA was performed by 3′ full RACE Core Set Ver. 2.0 (TaKaRa, Kyoto, Japan), 5′ full RACE Core Set Ver. 2.0 (TaKaRa, Kyoto, Japan) and SMARTer^TM^ RACE cDNA Amplification Kit (Clontech, Mountain View, CA, USA ), the specific operations were performed according to the manufacture′s guidelines. The first strand cDNA was synthesized from total RNA, and then the 3′ and 5′ ends of cDNAs were amplified with the designed gene-specific primers and the universal primers provided by the kits. In addition, PCR conditions were in accordance with request of kits and the annealing temperature of primers.

### 4.7. Gene Expression Analysis

Q-PCR was performed on a BIO-RAD CFX96^TM^ Real-Time System (C1000^TM^ Thermal Cycler) (Bio-Rad, Hercules, CA, USA). The RNA samples were quantified by spectrophotometer (Eppendorf, Hamburg, Germany) at the wavelength of 260 nm. The cDNA was synthesized from 1 μg RNA using PrimeScript^®^ RT reagent Kit With gDNA Eraser (TaKaRa, Kyoto, Japan). Q-PCR was carried out using the SYBR^®^ Premix Ex Taq^TM^ (Perfect Real Time) (TaKaRa, Kyoto, Japan) and contained 2 × SYBR Premix Ex Taq^TM^ 12.5 μL, 50 × ROX Reference Dye II 0.5 μL, 2 μL cDNA solution as a template, 1 μL mix solution of target gene primers and 9 μL ddH_2_O in a final volume of 25 μL. The amplification was carried out under the following conditions: 50 °C for 2 min followed by an initial denaturation step at 95 °C for 5 min, 40 cycles at 95 °C for 15 s, 51 °C for 15 s, and 72°C for 40 s. Gene relative expression levels were calculated by the 2^−ΔΔCt^ comparative threshold cycle (*C*t) method. The *C*t values of the triplicate reactions were gathered using the Bio-Rad CFX Manager V1.6.541.1028 software (Bio-Rad, Hercules, CA, USA).

### 4.8. Sequence and Statistical Analysis

Sequence retrieve was using the GenBank BLAST [46]. Sequence alignment and the phylogenetic tree were constructed by DNAMAN 5.0 and MEGA 5.05 [47], respectively. All data were means of three replicates at least with standard deviations. The results were analyzed for variance using the SAS/STAT statistical analysis package (version 6.12, SAS Institute, Cary, NC, USA, 1997). The difference between the means was tested by least significant difference at *P_0.05_* (LSD_0.05_). Figures were drawn by SigmaPlot 10.0 (SPSS Inc.: Chicago, IL, USA, 1999).

## Figures and Tables

**Table 1:** Morphological indices of development *Paeonia lactiflora*.

Development Stages	S1	S2	S3	S4
Plant height (cm)	49.63 ± 0.40 d	59.34 ± 0.13 c	64.93 ± 0.09 b	67.64 ± 0.09 a
Diameter of inflorescence stem (cm)	0.28 ± 0.01 c	0.47 ± 0.00 b	0.63 ± 0.00 a	0.64 ± 0.00 a
Fresh weight of inflorescence stem (g)	0.49 ± 0.08 c	0.78 ± 0.00 b	0.86 ± 0.00 a	0.86 ± 0.01 a
Diameter of flower (cm)	1.19 ± 0.02 d	2.00 ± 0.01 c	9.47 ± 0.10 b	12.61 ± 0.05 a
Fresh weight of flower (g)	0.69 ± 0.03 d	3.45 ± 0.08 c	18.65 ± 0.20 b	25.82 ± 0.04 a

Data mean values ± SE.

a,b,c,d indicate significant difference at the 0.05 level.

**Table 2:** Correlation of morphological indices and mechanical strength of inflorescence stem.

Morphological Indices	*R* (Correlation Coefficient)
Plant height (cm)	0.95 *
Diameter of inflorescence stem (cm)	0.98 *
Fresh weight of inflorescence stem (g)	0.88
Diameter of flower (cm)	0.96 *
Fresh weight of flower (g)	0.96 *

* indicates significant difference at the 0.05 level.

**Table 3:** Microstructure of inflorescence stem.

Development Stages	S1	S2	S3	S4
Number of vascular bundle	14.67 ± 1.53 c	25.33 ± 1.15 b	36.67 ± 1.53 a	38.33 ± 1.53 a
Vascular bundle (%)	22.40 ± 0.75 b	32.19 ± 0.48 a	39.09 ± 1.39 a	41.35 ± 2.33 a
Pith (%)	6.95 ± 1.07 b	7.82 ± 0.18 b	12.06 ± 1.26 a	14.91 ± 2.13 a

Data mean values ± SE.

a,b,c indicate significant difference at the 0.05 level.

**Table 4:** Primers used for genes isolation and expression pattern.

Primer	Oligonucleotide Sequence (5′-3′)	Application
*CA3-1*	GAGCTGCTATGTGTCTGATGA	1st of 3′ RACE
*CA3-2*	GAGTTTGCAAGGAGATGGGT	2nd of 3′ RACE
*CA3-3*	CCCCTCCTCTATATCCTCCAGGCTGAA	5′ RACE
*CA6-1*	TTGTGAAAGAACGGAGGG	1st of 3′ RACE
*CA6-2*	TGCCAAGGCTCAAAAGGT	2nd of 3′ RACE
*CA6-3*	TTCTTGTTGGTGGCTTCT	1st of 5′ RACE
*CA6-4*	TCATACTCTCTCTTCATTGCCC	2nd of 5′ RACE
*CCoAOMT1*	TTGTGAAAGAACGGAGGG	1st of 3′ RACE
*CCoAOMT2*	TGCCAAGGCTCAAAAGGT	2nd of 3′ RACE
*CCoAOMT3*	CCCATTCCATAGGGTGTTGTCGTAGCC	5′ RACE
*Actin_F_*	GCAGTGTTCCCCAGTATT	expression pattern analysis
*Actin_R_*	TCTTTTCCATGTCATCCC	
*CA3_F_*	GTTGCCTCTACGCTTATG	
*CA3_R_*	CACTTCCCCACTCTGATT	
*CA6_F_*	GGGTTATTGAAGGTTTTAGC	
*CA6_R_*	TATCAGCAGTGTAGTCGGA	
*PAL_F_*	ACATTCTCGCCACTACCA	
*PAL_R_*	CTTCCGAAATTCCTCCAC	
*CCoAOMT_F_*	GCGTGAAGTAACAGCAAAAC	
*CCoAOMT_R_*	AGAGCAGTAGCAAGGAGAGA	
